# Clinical Outcome after Colonic Resection in Women with Endometriosis

**DOI:** 10.1155/2015/514383

**Published:** 2015-07-15

**Authors:** Bettina Klugsberger, Andreas Shamiyeh, Peter Oppelt, Christina Jabkowski, Wolfgang Schimetta, Dietmar Haas

**Affiliations:** ^1^Second Surgical Department, Ludwig Boltzmann Institute for Surgical Laparoscopy, Academic Teaching Hospital, Linz General Hospital, 4021 Linz, Austria; ^2^Faculty of Medicine, Johannes Kepler University, 4040 Linz, Austria; ^3^Department of Obstetrics and Gynecology, Women's General Hospital, 4020 Linz, Austria; ^4^Department of Gynecology, Erlangen University Hospital, 91054 Erlangen, Germany; ^5^Department of Applied Systems Research and Statistics, Johannes Kepler University, 4040 Linz, Austria

## Abstract

*Background*. In severe forms of endometriosis, the colon or rectum may be involved. This study evaluated the functional results and long-term outcome after laparoscopic colonic resection for endometriosis. * Patients and Methods.* Questionnaire survey with 24 women who had experienced typical symptoms, including pelvic pain, infertility, and endometriotic lesions in the bowel and undergone laparoscopic surgery, including low anterior resection, from 2009 to 2012, was conducted. * Results.* Information about the postoperative outcome was obtained from 22 women and was analyzed statistically. Twenty-one had undergone low anterior resection; one patient required a primary Hartmann procedure due to a rectovaginal fistula. The conversion rate was 4.5%. Major complications occurred in one patient, including an anastomotic leakage, and a Hartmann procedure was carried out subsequently in this patient. The symptoms of pain during defecation, pelvic pain, dyspareunia, dysmenorrhea, and hematochezia showed clear improvement one year after the operation and at the time of the questionnaire. * Conclusion*. Laparoscopic low anterior resection for deeply infiltrative endometriosis is technically demanding but feasible and safe, and it improves the clinical symptoms of endometriosis in the bowel.

## 1. Introduction

Endometriosis is one of the most frequent benign diseases and can affect 7–15% of women of reproductive age [1]. The condition is defined as the presence of endometrial glands and stroma outside the uterus. These ectopic endometrial implants are usually located in the pelvis but can occur almost anywhere in the body [2]. In severe forms of the condition, the colon or rectum may be involved in approximately 25% of cases [3]. Depending on the menstrual cycle, endometriosis may cause clinical symptoms such as pain, functional disorders, and infertility. Surgical removal of the affected tissue is the “gold standard” treatment in these cases [4].

No guidelines or standardized recommendations are available on the surgical approach in patients with deeply infiltrating endometriosis. The therapeutic strategies that have been described (e.g., shaving, disk resection, and segmental resection) are inconsistent [5–10], as is the definition of this specific form of endometriosis itself. The revised version of the Enzian classification may help quantify and define the disease further [11].

The aim of the present study was to evaluate the functional results and long-term outcome after laparoscopic colonic resection for endometriosis.

## 2. Patients and Methods

**Figure 1:**
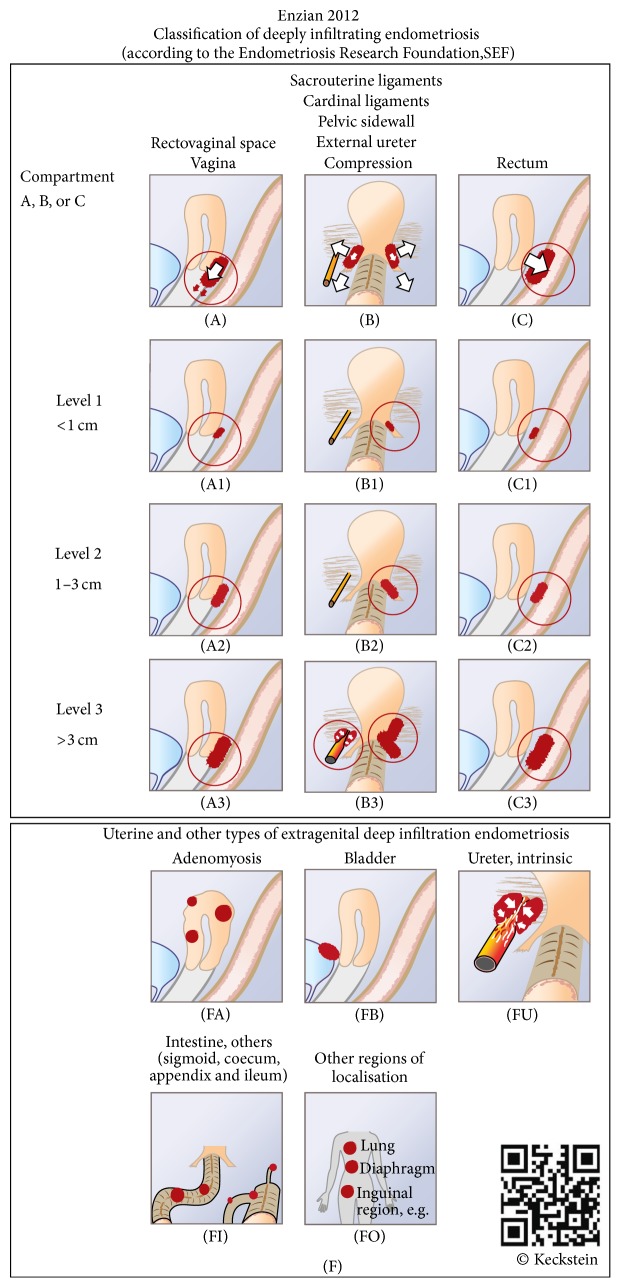
The Enzian classification.

Approval for the study from the local institutional ethics committee was obtained on April 9, 2014 (ref. numberL-17-14). An analysis was carried out of the data for 24 patients who had undergone laparoscopic rectal resection for deeply infiltrating endometriosis between January 1, 2009, and December 31, 2012, at the Departments of General Surgery and Gynecology at the General Hospital in Linz, Austria. Endometriosis was confirmed histologically after laparoscopic surgery. All of the operations were carried out by the same team of four visceral surgeons (each of whom had previously carried out at least 100 laparoscopic colonic resections) and four gynecologists (each of whom had previously carried out at least 200 laparoscopies). The patients were classified postoperatively using the Enzian classification (Figure 1).

Twenty-two of the patients were interviewed postoperatively by phone in May 2014 by a gynecologist; two patients could not be reached by phone. A questionnaire was filled out for each patient, recording responses on intestinal and gynecological symptoms: presence of pain associated with menstruation, diarrhea, constipation, hematochezia, dyspareunia, and dysmenorrhea. The responses—scored using a numerical rating scale from 0 (absent) to 10 (unbearable)—were documented before surgery, 1 year after surgery, and at time of the phone survey. All data concerning the operations and changes resulting from it were reported. Informed consent was obtained from all of the patients.

Criteria for inclusion in the study were symptomatic deeply infiltrating endometriosis with histological confirmation, age over 18 years, and legal capacity. Irregularities in the study protocol and absence of a consent form were exclusion criteria.

All of the women underwent a bimanual rectovaginal examination, vaginal ultrasonography, and colonoscopy. The indication for surgery was established by the gynecologist on the basis of intestinal stenosis associated with intestine-related symptoms. Complete laparoscopic management was planned in all of the patients, including resection of all visible endometriotic lesions. On the basis of the intraoperative findings, bowel resection was performed if there was deep invasion of the bowel—that is, with infiltration at least into the muscularis mucosae.

The laparoscopic procedure was performed with the patient in the Lloyd-Davies position. The operation was started by the gynecologist. Access was via the umbilical port (11 mm), with peritoneal insufflation with CO_2_ gas to the pressure of 12 mm Hg. After insertion of the laparoscope (Storz, Germany), additional ports were established under direct vision—one suprapubic (12 mm), one in the right iliac fossa (5 or 12 mm), and one in the left iliac fossa (5 mm). After exploration of the pelvic cavity, adhesiolysis and ovarian cystectomy were performed if necessary. All endometriotic lesions were excised.

Low anterior resection (LAR) of the rectum was necessary in some cases, and the operation was then continued by the general surgeon. The colorectum was mobilized and the ureter was visualized on the left side. The pouch of Douglas (rectouterine pouch) was opened, and the rectum was released from the mesorectal tissue before the colorectum was separated caudal to the endometriotic nodule using an endostapler. Depending on the size of the affected bowel, the colorectum was withdrawn through a small 3–5 cm Pfannenstiel incision and resected as much as needed. Dissection was carried out with monopolar scissors and the LigaSure Atlas device (LigaSure 5 mm Blunt Tip 44 cm; Covidien Austria Ltd., Brunn am Gebirge, Austria).

The anvil of a circular stapling device (Touchstone, 29 mm; Dach Medical Group, Bürmoos, Austria) was inserted into the descending colon, which was then returned to the peritoneal cavity. The incision was closed with running sutures and the anastomosis was carried out laparoscopically with the circular stapling technique. Intraoperative endoscopic assessment of the anastomosis was performed.

### 2.1. Statistical Analysis

For intraindividual comparisons between the three time points, Friedman's exact rank sum test was used for metric variables, none of which had normally distributed data sets, followed by Schaich-Hamerle multiple comparisons. Dichotomous variables were compared using the exact Cochran-*Q* test, followed by multiple comparisons with exact McNemar tests with Bonferroni correction. The only comparison of a dichotomous variable between two time points was performed using the exact McNemar test.

For subgroup analysis, the* t*-test for independent samples was used to compare metric variables with normally distributed data sets; the exact Mann-Whitney *U* test was used to compare metric variables without normally distributed data sets and the only variable that was measured on an ordinal scale; and Fisher's exact test was used to compare dichotomous variables.

Forward stepwise multiple regression analyses were carried out to detect preoperative factors influencing pain during defecation and dyspareunia. The same independent variables were used in both regression analyses: age (years), time of follow-up (month), body mass index (kg/m^2^), size of endometriotic lesion (cm), duration of surgery (minutes), duration of gynecological surgery (minutes), total duration of surgery (minutes), pain during defecation preoperatively, diarrhea preoperatively, constipation preoperatively, lower abdomen pain preoperatively, dyspareunia preoperatively, dysmenorrhea preoperatively, menstrual-related pain during defecation preoperatively, hematochezia before surgery, preoperative findings in the abdomen, and Enzian classifications of the C1, C2, and C3 compartments.

Type I error was not adjusted for multiple testing. The results of the inferential statistics are therefore only descriptive. Statistical analysis was performed using the open-source* R* statistical software package, version 3.0.2.

Data are presented as means ± standard deviation (SD).

## 3. Results

After a median follow-up period of 42.4 ± 14.04 months, 22 women were contacted for a phone interview. Two women could not be reached by phone. The patients' mean age was 35.9 ± 6.21 years. Seven patients (31.8%) reported that they had had a pregnancy since the time of the surgery. The patients' mean body mass index (BMI, calculated as weight in kilograms divided by the square of height in meters) was 22.5 ± 3.0. The mean operating time was 287.2 ± 100.09 min (Table 1). Nine patients (40.9%) had previously undergone abdominal surgery.

Twenty-one patients received low anterior resection; the conversion rate was 4.5% (1/21). One patient required a primary Hartmann procedure due to a rectovaginal fistula. Major complications occurred in one patient, including an anastomotic leakage. A Hartmann procedure was carried out subsequently in this patient (Table 2).

Nine patients (40.9%) reported hematochezia preoperatively; two patients (9.1%) reported hematochezia 1 year after the operation (*P* = 0.004). Eleven patients (50%) reported alterations in the stool form after surgery. The preoperative values reported for the intensity of diarrhea and constipation (on a scale of 0 = absent to 10 = unbearable) were 1.82 ± 3.16 and 3.09 ± 3.94, respectively. One year after surgery, the reported values for the intensity of diarrhea and constipation were 1.95 ± 2.85 and 2.23 ± 3.21, respectively. No improvement was seen over time with regard to diarrhea and constipation, with overall *P* values of 0.885 and 0.825, respectively.

A total of seven patients (31.8%) were receiving hormonal therapy before surgery, compared with eight patients (36.4%) 1 year postoperatively. Before surgery, 15 patients (68.2%) reported menstrual related pain during defecation, with a mean pain value (on a scale of 0–10) of 6.77 ± 3.57. The mean value for preoperative pelvic pain was 8.18 ± 2.87. Two patients (9.1%) reported menstrual related pain during defecation 1 year after the operation, with mean pain values at that time of 1.41 ± 2.44. The mean score for pelvic pain 1 year after surgery was 2.95 ± 2.98. There was also clear improvement with regard to dyspareunia and dysmenorrhea. The clinical symptoms reported by the patients before surgery, 1 year after surgery, and at the time of the phone survey are summarized in Table 3.

All of the patients had lesions in compartment C (rectum and sigmoid). One patient (9.1%) was classified as C1, nine patients (40.9%) were classified as C2, and 12 patients (54.5%) were classified as C3. The mean size of the endometriotic lesions was 2.86 ± 1.30 cm.

Regression analysis showed that dyspareunia 1 year after surgery can be predicted using a model including preoperative dyspareunia, age, and endometriotic lesions in compartment C2 (adjusted *R* ^2^ = 0.628) (see Supplemental Digital Content 1 in Supplementary Material available online at http://dx.doi.org/10.1155/2014/514383).

A subgroup analysis was carried out to check potential errors related to the time of the questionnaire. The patients were divided into two subgroups: median follow-up period >46 months (*n* = 11) and median follow-up period ≤46 months (*n* = 11). However, there was no evidence that the interval between the operation and the survey had any effect on the results (Supplemental Digital Content 2).

## 4. Discussion

Surgical therapy in patients with deeply infiltrating endometriosis of the bowel may be associated with highly morbid conditions including rectovaginal fistula, temporary or definitive artificial anus, and anastomotic insufficiency [12], in addition to considerable blood loss, blood transfusion requirements, and a need to convert the procedure to a laparotomy [13–17]. However, several studies have reported that colorectal resection for deeply infiltrating endometriosis is a safe and effective procedure, with an acceptable rate of postoperative complications and that it significantly improves the patients' quality of life [18, 19].

In the present study, considerable improvements were observed postoperatively in pain-related symptoms such as pain during defecation, dysmenorrhea, dyspareunia, pelvic pain, and hematochezia. Postoperative pregnancy rates of 45–48% have been reported in the literature [20]. The rate was 31.8% in the present study, but none of the patients underwent preoperative fertility examinations and the extent to which they made use of assisted reproduction services afterwards was not investigated.

The anastomotic leakage rate was 4.5% and the conversion rate was also 4.5% in the present study. Jelenc et al. reported a conversion rate of 5.4% and an anastomotic leakage rate of 5.8% (3/52) [12]. The results are therefore comparable with those in the literature, although the small number of patients included is a major limitation of the study. Despite this, there was a notable improvement in the patients' clinical symptoms.

It is also notable that the marked postoperative improvement in symptoms that was observed showed no major differences between the two time points of 1 year postoperatively and the time of the questionnaire survey. This may suggest that sustained improvement occurs and is maintained even several years after surgery. This hypothesis is supported by the subgroup comparison of the questionnaire time point, which showed that the time point had no significant influence.

## 5. Conclusions

The present study shows that laparoscopic low anterior resection in patients with deeply infiltrating endometriosis of the bowel has positive effects on pain during defecation, dysmenorrhea, dyspareunia, pelvic pain, and hematochezia 1 year after surgery and also later. Laparoscopic low anterior resection is a technically demanding but feasible and safe procedure, and these results suggest that it improves the clinical symptoms of endometriosis in the bowel and the fertility rate.

## Supplementary Material

SUPPLEMENTAL DIGITAL CONTENT 1: Regression analysis shows that dyspareunia 1 year after surgery can be predicted by a model including preoperative dyspareunia, age and endometriotic lesions in compartment C2 (adjusted R2 = 0.628).
SUPPLEMENTAL DIGITAL CONTENT 2: A subgroup analysis was carried out to check potential errors related to the time of the questionnaire. The patients were divided into two subgroups: median follow-up period > 46 months (n = 11) and median follow-up period ≤ 46 months (n = 11).

## Figures and Tables

**Table 1:** Demographic and clinical characteristics in the 22 patients.

Characteristics	Mean ± SD
Age (years)	35.9 ± 6.2
BMI	22.5 ± 3.0
Follow-up (months)	42.4 ± 14.0
Size of endometrial lesion (cm)	2.9 ± 1.3
Operating time (minutes)	287.2 ± 100.1

BMI: body mass index.

**Table 2:** Types of surgery carried out.

Operation	Patients (*n*)
Laparoscopic low anterior resection	21
Conversion to open procedure	1
Anastomotic leak, Hartmann procedure	1
Primary Hartmann procedure in a case of rectovaginal fistula	1

**Table 3:** Patients' clinical symptoms before surgery, 1 year postoperatively, and at the time of the phone survey.

Characteristics	Preoperative	1 year postoperatively	At time of telephone survey	*P* ^a^	*P* ^b^	*P* ^c^	*P* ^t^
Pain during defecation^1^	6.77 ± 3.57	1.41 ± 2.44	1.91 ± 2.81	<0.001^**^	0.007^**^	<0.903	<0.001^**^
Diarrhea^1^	1.82 ± 3.16	1.95 ± 2.85	1.5 ± 2.15				0.885
Constipation^1^	3.09 ± 3.94	2.23 ± 3.21	2.41 ± 3.11				0.825
Pelvic pain^1^	8.18 ± 2.87	2.95 ± 2.98	2.09 ± 2.96	0.001^**^	<0.001^**^	0.528	<0.001^**^
Dyspareunia^1^	3.18 ± 3.45	1.64 ± 2.54	1.86 ± 2.46	0.147	0.169	0.997	0.003^**^
Dysmenorrhea^1^	8.00 ± 3.10	2.45 ± 3.00	2.73 ± 3.37	<0.001^**^	<0.001^**^	0.997	<0.001^**^
Hematochezia (yes/no)				0.004^**^	0.004^**^	>0.999	<0.001^**^

Data are presented as means plus or minus standard deviation.

^1^Numerical rating scale: the question can be answered with 0 (absent) to 10 (unbearable).

*P* ^t^: *P* value overall; *P* ^a^: *P* value before surgery versus 1 year postoperatively; *P* ^b^: *P* value before surgery versus time of telephone survey; *P* ^c^: *P* value 1 year postoperatively versus time of telephone survey. ^*^ *P* < 0.05; ^**^ *P* < 0,01.

## Abbreviations

LAR: Laparoscopic low anterior resection
SD: Standard deviation.
