# Supplementary material for: Clinical Outcome after Colonic Resection in Women with Endometriosis
Source: Biomed Res Int. 2015 Jul 15;2015:514383. doi: 10.1155/2015/514383 (PMC4518181; doi:10.1155/2015/514383)
Supplement: Supplementary file 1 — SUPPLEMENTAL DIGITAL CONTENT 1: Regression analysis shows that dyspareunia 1 year after surgery can be predicted by a model including preoperative dyspareunia, age and endometriotic lesions in compartment C2 (adjusted R2 = 0.628). SUPPLEMENTAL DIGITAL CONTENT 2: A subgroup analysis was carried out to check potential errors related to the time of the questionnaire. The patients were divided into two subgroups: median follow-up period > 46 months (n = 11) and median follow-up period ≤ 46 months (n = 11). [file 514383.f1.docx]

Supplemental Digital Content 1: Regression analysis shows that dyspareunia 1 year after surgery can be predicted by a model including preoperative dyspareunia, age and endometriotic lesions in compartment C2 (adjusted *R^2^* = 0.628).

| Dyspareunia 12 months postoperatively | Nonstandardized coefficient | *P* |
| --- | --- | --- |
| (Constant) | –5.110 |  |
| Preoperative dyspareunia | 0.429 | 0.001** |
| Age (y) | 0.132 | 0.026* |
| Enzian classification of the C2 compartment | 1.558 | 0.050 |

* *P* < 0.05; ** *P* < 0,01

Supplemental Digital Content 2: A subgroup analysis was carried out to check potential errors related to the time of the questionnaire. The patients were divided into two subgroups: median follow-up period > 46 months (n = 11) and median follow-up period ≤ 46 months (n = 11).

| Parameter | Test | *P* |
| --- | --- | --- |
| Age (y) | MWU | 0.548 |
| BMI (kg/m^2^) | *t*-test | 0.714 |
| Size of endometriotic lesion (cm) | MWU | 0.414 |
| Total operating time (min) | MWU | 0.073 |
| Pain during defecation |  |  |
| Preoperatively | MWU | 0.291 |
| 12 months postoperatively | MWU | 0.291 |
| At time of questionnaire survey | MWU | 0.186 |
| Diarrhea |  |  |
| Preoperatively | MWU | 0.408 |
| 12 months postoperatively | MWU | 0.329 |
| At time of questionnaire survey | MWU | 0.085 |
| Constipation |  |  |
| Preoperatively | MWU | 0.688 |
| 12 months postoperatively | MWU | 0.524 |
| At time of questionnaire survey | MWU | 0.442 |
| Lower abdominal pain |  |  |
| Preoperatively | MWU | > 0.999 |
| 12 months postoperatively | MWU | 0.106 |
| At time of questionnaire survey | MWU | 0.323 |
| Dyspareunia |  |  |
| Preoperatively | MWU | 0.621 |
| 12 months postoperatively | MWU | 0.042* |
| At time of questionnaire survey | MWU | 0.519 |
| Dysmenorrhea |  |  |
| Preoperatively | MWU | 0.055 |
| 12 months postoperatively | MWU | 0.222 |
| At time of questionnaire survey | MWU | 0.078 |
| Menstrual-related pain during defecation |  |  |
| Preoperatively | Fisher | > 0.999 |
| 12 months postoperatively | Fisher | > 0.999 |
| At time of questionnaire survey | Fisher | 0.635 |
| Hematochezia |  |  |
| Preoperatively | Fisher | > 0.999 |
| 12 months postoperatively | Fisher | > 0.999 |
| At time of questionnaire survey | Fisher | > 0.999 |
| Contraceptive pill use |  |  |
| Preoperatively | Fisher | > 0.999 |
| 12 months postoperatively | Fisher | > 0.999 |
| At time of questionnaire survey | Fisher | > 0.999 |
| Altered form of feces |  |  |
| 12 months postoperatively | Fisher | 0.395 |
| At time of questionnaire survey | Fisher | 0.387 |
| Successful pregnancy between surgery and time of questionnaire survey | Fisher | > 0.999 |
| Prior abdominal surgery | Fisher | > 0.999 |
| Enzian classification of the C compartment | MWU | > 0.999 |

* *P* < 0.05

Fisher, Fisher’s exact test; MWU, exact Mann–Whitney U test; *t-test, t-test for independent samples*
